# ChatGPT assisted generation of systematic review ideas in urooncology

**DOI:** 10.1590/1806-9282.20250910

**Published:** 2026-06-15

**Authors:** Ahmet Emin Dogan, Gorkem Ozenc

**Affiliations:** 1 Etlik City Hospital – Ankara, Türkiye.

**Keywords:** Generative artificial intelligence, Research design, Systematic reviews as topic, Urologic neoplasms

## Abstract

**OBJECTIVE:**

The aim of this study was to analyze the performance of ChatGPT in the generation of new systematic review ideas in urooncology.

**METHODS:**

In September 2024, we requested ChatGPT Version 4.0 to generate 10 systematic review ideas in general urooncology and also 10 ideas for each of the four subcategories: bladder cancer, prostate cancer, renal cell carcinoma, and testicular cancer. We utilized PubMed and Scopus to examine 50 ideas to determine whether prior systematic reviews had addressed them. Novelty was defined as a topic without prior systematic review.

**RESULTS:**

ChatGPT generated 30% original systematic review ideas, with 15 out of 50 being novel. The novelty rate for general urooncology was 50%. The rates for subcategories were 10% for both bladder and prostate cancer, 50% for renal cell carcinoma, and 30% for testicular malignancies. Approximately 10% of general research concepts external to systematic reviews were novel.

**CONCLUSION:**

ChatGPT performed very well in producing creative, apt, and partially viable systematic review ideas in urooncology. Although human judgment is still required to determine feasibility and create proposals with better accuracy, ChatGPT and other large language models can be useful aids while designing research, especially for dynamic and information-heavy disciplines such as urooncology.

## INTRODUCTION

The use of the internet and artificial intelligence (AI)-supported information systems is increasing in various fields, including medicine. Computerization and the use of robotics for human-machine intelligence are driving a paradigm shift in healthcare delivery^1^. Researchers use these systems due to the ease of accessing information. Generative AI (GenAI), a subset of AI, is especially widespread because of its ability to create new outputs independent of predefined instructions^2^. In addition to GenAI, AI platforms such as OpenAI’s ChatGPT and Google’s Gemini have shown promising applications in healthcare. It has been evaluated that ChatGPT provides sufficient and largely accurate answers to medical inquiries^3^. This ability, added to its non-judgmental and non-human character, makes it especially well-suited to discussing delicate issues like sexuality and reproductive health^4,5^. ChatGPT can both answer questions posed by researchers and assist in generating innovative ideas^6,7^. In addition, AI has been reported to improve the quality and readability of scientific writing^8^. Recent reviews have chronicled both ChatGPT’s potential and ethical challenges in medicine, highlighting its wide areas of application and methodological openness requirements^9,10^.

Continuing research in urooncology is crucial to enhance evaluation, treatment, and outcomes in patients. Previously, researchers have employed numerous strategies, including literature review, expert opinion, clinical practice, and conference publications, to describe areas of documented knowledge gaps and develop research plans. The exponentially growing number of scientific papers and the interdisciplinarity of urooncology research have rendered it progressively more difficult to detect knowledge gaps. It has been emphasized before that it is necessary to take the time to develop a good question for a study^11,12^. Good research questions are not only important for individual manuscripts but also for research synthesis, for example, systematic review (SR) and meta-analysis, which usually involve a quality appraisal of included studies^13^.

This study aligns with the complementary cognition framework, which highlights the synergy between human contextual reasoning and AI’s rapid pattern recognition^14^. ChatGPT-4.0 was selected for its demonstrated accuracy in medical information tasks^3^ and its superior evaluator scores for accuracy and overall quality compared with Google Gemini and Anthropic Claude in a recent benchmarking study on medical content generation^15^, while maintaining broad accessibility for reproducible academic research.

The current data is toward comparing the results of large language models (LLMs) to human performance. An SR and meta-analysis by Takita et al. was able to show that generative AI models are able to estimate physician-level diagnostic accuracy in multiple areas, albeit with considerable variation present^16^. This study emphasizes the requirement to have a baseline from a human while measuring originality or usability of ChatGPT-generated ideas.

The purpose of this study was to evaluate the capacity of ChatGPT as an adjunctive tool for the generation of novel SR ideas in urooncology and to establish its usefulness in the facilitation of research planning.

## METHODS

We carried out a descriptive cross-sectional study in September 2024 on ChatGPT Version 4.0 via the official OpenAI platform (chat.openai.com).

To facilitate immediate standardization and replicability, the complete battery of questions was entered into ChatGPT-4.0 via the official web interface. Test sessions were started within a fresh chat window in an effort to suppress context memory effects from prior interaction.

A standardized prompt was used for each field: “Create 10 novel SR ideas that have not been published in the field of general urooncology, bladder cancer, prostate cancer, renal cell carcinoma, or testicular cancer. Avoid topics that already have extensive existing reviews.” Subfields were selected based on their frequency of occurrence in urooncology practice. Statistical results of novelty creation are presented in Table 1, both overall and by subcategory, stratified. This prompt was designed to balance creativity with clinical relevance as well as limit investigator-driven optimization. The prompt was not iteratively improved or optimized, as the main goal was simply to test the basic creativity of ChatGPT under standardized conditions and not its potential maximal performance.

**Table 1:** Novelty and publication density of ChatGPT-suggested systematic review ideas in urooncology.

Subcategories	Median, Q1–Q3		Total publications (all ideas)		Novelty accuracy rate, 95%CI		NNI	
	SR	GR	SR	GR	SR	GR	SR	GR
General urooncology	7 6–17	18 5–103.5	57	419	50.0% 23.7–76.3	20.0% 5.7–51.0	7.14	1.11
Bladder cancer	4 1–7	12 6–109	56	517	10.0% 1.8–40.4	0% 0–27.8	2.50	0
Prostate cancer	9 6–10	41 28–94	84	589	10.0% 1.8–40.4	10.0% 1.8–40.4	1.11	0.24
Renal cell carcinoma	3 2–11	10 6–50	32	306	50.0% 23.7–76.3	20.0% 5.7–51.0	16.7	2.00
Testicular cancer	2 1.5–4	14 7–74.5	24	379	30.0% 10.8–60.3	0% 0–27.8	15.0	0
Overall	6 2.5–10	14 5–67.5	253	2,210	30.0% 19.1–43.8	10.0% 4.3–21.4	–	–

CI: confidence interval; GR: general research; NNI: normalized novelty index; Q1–Q3: interquartile range (25th–75th percentile); SR: systematic review.

The temperature parameter was set to 0.7 to make room for imagination as well as for relevance of the topic, and these top_p parameters were set to 1.0. Other parameters, such as frequency penalty and presence penalty, were set to their default.

We searched the PubMed and Scopus databases for 50 research ideas obtained from ChatGPT. Ideas were considered “novel SR concepts” if no SR on the topic was identified in PubMed or Scopus from January 1, 1990, to October 1, 2024. We compared the novelty of ideas generated by ChatGPT with publications in PubMed and Scopus up to October 1, 2024. To be open about methodology, we labeled any SRs published since October 2023—ChatGPT-4.0’s reported knowledge cut-off—as “retroactively novel” because the AI had no possibility of being aware of those publications. Those instances were counted separately in a sensitivity analysis. Where there was no SR of a topic but other types of publication were present, the idea was classified as “general research” (GR). Themes unable to identify even a single publication were categorized as “unique GR concepts.” In our study, we classified these unique topics created by ChatGPT as new under the title of GR. We employed the identical methodology for overarching research subjects.

To quantify the stability of output, each prompt was executed twice; if two results were very different in content (i.e., greater than 50% topic change), a third run was performed. Of these, the first consistent, medically sound set of 10 ideas was chosen for verification. All outputs were manually verified for urooncology relevance, conceptual coherence, and novelty within the dataset.

In addition to bibliometric database screening, to resolve concept novelty aside from bibliographic replication, two authors independently reviewed all the topics provided by AI and agreed on their clinical importance and research relevance. Expert opinion was also obtained from a well-experienced urologist (Dr. Adem Sanci, Department of Urology, Etlik City Hospital). All 50 suggestions provided by ChatGPT were therefore assessed and categorized, on the basis of Scopus and PubMed searching and subject matter expert opinion, as being “novel” or “already published” for SR and GR scenarios. Expert adjudication was implemented as a supporting qualitative step to validate the clinical relevance, conceptual consistency, and urooncological suitability of AI-generated topics and to exclude recommendations that were manifestly trivial or thematically irrelevant. Bibliographic novelty, as determined by PubMed and Scopus searches, remained the primary criterion and was not overridden by expert review. The evaluation process was consensus-based and descriptive; therefore, a formal inter-rater reliability metrics were not calculated. To put novelty rates into context across subfields, we additionally noted the overall number of SRs published across each category (bladder, prostate, renal, and testicular cancer) in the period January 1990 to October 2024, utilizing PubMed and Scopus. By doing so, we could determine if lowered novelty rates in certain categories (prostate cancer, for instance) might be indicative of publication saturation rather than generation capacity limitations in ChatGPT. Novelty rates were reported with 95%CIs calculated by the Wilson score method to deal with small sample binomial data.

To control for publication density within subfields, we also computed a Normalized Novelty Index (NNI). We performed this calculation as: NNI=(Novelty rate, %)/(Median number of pre-existing SRs for non-novel ideas). For GR concepts, the parallel estimate was calculated using the same formula but substituting SRs with earlier GR literature. This is to enable comparison of novelty rates, controlling for heterogeneity in baseline publication density between cancer subtypes.

Five prototypic SR questions—one for each of the five categories—were chosen according to clinical importance to typical urooncology practice, apparent conceptual originality by expert peer assessment, and suitability for SR implementation in typical methodological designs. These topics included the impact of molecular subtyping on personalized treatment in urothelial carcinoma, the role of gut microbiota in renal cell carcinoma progression and treatment response, the influence of environmental pollutants on bladder cancer incidence, psychosocial and mental health outcomes in testicular cancer survivors, and the cognitive effects of androgen deprivation therapy in prostate cancer.

Duplicate entries were found and removed using DOI (digital object identifier)-based matching to guarantee that every suggested topic is distinct. Novelty accuracy rates of SR and GR were computed as the ratio of new ideas produced by ChatGPT to the total SR and GR publications. Novelty accuracy rates overall for general SR and GR were also calculated. Continuous variables were summarized using medians and interquartile ranges (IQRs). Median and IQR were calculated for non-novel ideas only, as novel ideas, by definition, had zero previous SRs (SR count=0), and their inclusion would have shifted the distribution to zero. ChatGPT was used merely for the generation of research ideas based on prompts developed and not for writing or editing the manuscript.

## RESULTS

ChatGPT had a cumulative SR novelty accuracy of 30%, as 15 of 50 SR suggestions were novel. The other 35 topics (70%) were for previously published SRs. Of the non-novel ideas, the median number of previously published SRs per idea was 6 (IQR: 2.5–10). In a subanalysis by topic, ChatGPT created novel SR topics in 50% of instances for general urooncology. Non-novel topics for general urooncology had a median of 7 (IQR: 6–17) published corresponding SRs (Table 1).

For specific topics within urooncology, 25% of SR ideas proposed by ChatGPT were determined to be novel. Specifically, ChatGPT’s SR novelty accuracy rate was 10% for bladder cancer, 10% for prostate cancer, 50% for renal cell carcinoma, and 30% for testicular cancer. Non-novel ideas for specific topics had a median of 4 (IQR: 1–7), 9 (IQR: 6–10), 3 (IQR: 2–11), and 2 (IQR: 1.5–4), respectively (Table 1).

When looking at GR publications other than SRs, we determined that 10% of topics proposed by ChatGPT were novel without prior studies published on the suggested topic. ChatGPT demonstrated a GR novelty accuracy rate of 20, 10, and 20% for general urooncology, prostate cancer, and renal cell carcinoma, respectively. For non-novel GR ideas, the median number of previously published studies per idea was 18 (IQR: 5–103.5) for general urooncology, 12 (IQR: 6–109) for bladder cancer, 41 (IQR: 28–94) for prostate cancer, 10 (IQR: 6–50) for renal cell carcinoma, and 14 (IQR: 7–74.5) for testicular cancer. ChatGPT did not generate any novel GR ideas for bladder cancer or testicular cancer (Table 1).

For every subfield, novelty rates with 95%CIs are presented to provide more precision of the estimates (Table 1). The largest intervals were in general urooncology and renal cell carcinoma (95%CI 23.7–76.3, both), and then in testicular cancer (95%CI 10.8–60.3). Intervals for bladder and prostate cancer were smaller, indicating fewer new ideas in these diseases.

After controlling for saturation of publications in the subfield, we calculated NNI for the SR topics. This comparison proved that testicular cancer (NNI=15.0) and renal cell carcinoma (NNI=16.7) were most novel when adjusted, with general urooncology being next (NNI=7.14), followed by prostate cancer (NNI=3.33) and bladder cancer (NNI=2.50). These results show that the lower raw percentages of novelty for these areas, such as bladder and prostate cancer, are at least partially due to high saturation of prior SRs.

The same analysis was carried out for the GR topics with the same methodology. The highest adjusted novelty in this case was found for renal cell carcinoma (NNI=2.00) and general urooncology (NNI=1.11), and the lowest adjusted novelty was for prostate cancer (NNI=0.24), bladder cancer (0), and testicular cancer (0). This implies that novelty generation assisted with AI could do better in areas of moderate literature density, that is, RCC, but less so in densely covered areas (e.g., prostate, bladder) and sparse areas (e.g., testicular cancer).

**Figure 1:**
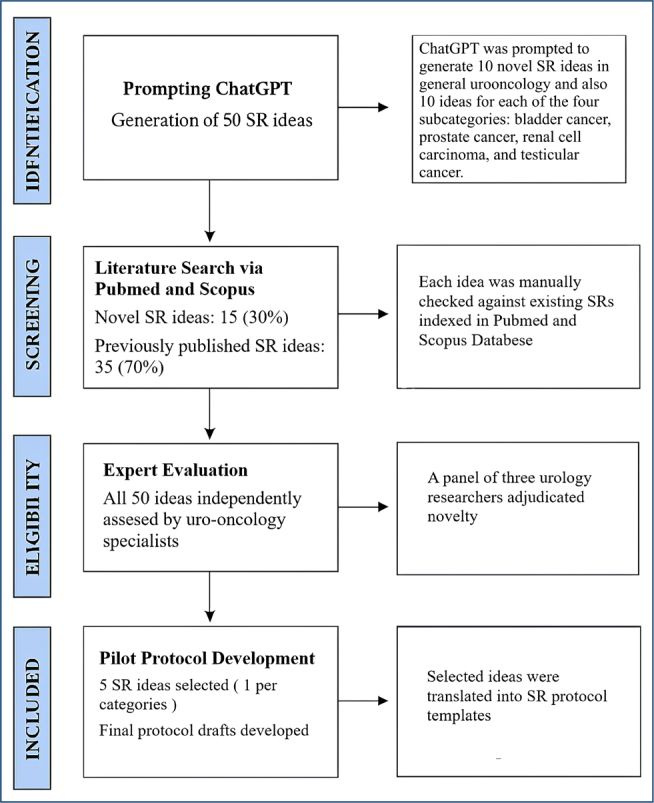
Flowchart of the generation and evaluation of systematic review ideas.

Information about the five representative ideas fulfilling all the pre-determined selection criteria is presented in Table 2. Table 2 shows in-depth protocol plans to demonstrate how such AI-derived concepts are effectively translated into evidence-based research planning. All topics were selected on the basis of clinical salience, perceived novelty, and suitability for SR execution. These examples demonstrate the potential usefulness of ChatGPT-generated concepts as an input for formal evidence synthesis in urooncologic research. Figure 1 provides a flow diagram of the study.

**Table 2:** Pilot protocols based on ChatGPT-generated systematic review ideas.

Subcategories	Systematic review title	Research question	Objectives	Outcomes
General urooncology	Impact of molecular subtyping on personalized treatment in urothelial carcinoma	How does molecular subtyping affect treatment decisions and outcomes in urothelial carcinoma patients?	To synthesize existing evidence on the role of molecular classification in treatment stratification for urothelial cancer.	Response rate, PFS, overall survival, adverse events
Bladder cancer	Sex and gender differences in bladder cancer	Are there molecular or clinical outcome differences between sexes in bladder cancer patients?	To explore biological and treatment-related disparities in men versus women.	Incidence, mortality, response to BCG or chemotherapy
Prostate cancer	Comparative efficacy of active surveillance versus radical prostatectomy in younger men with low-risk prostate cancer	Among younger men (<55 years) with low-risk prostate cancer, which approach yields better long-term outcomes?	To compare active surveillance versus surgery in terms of oncologic and quality-of-life outcomes.	OS, CSS, biochemical recurrence, QoL scores (sexual function, urinary continence)
Renal cancer	The role of gut microbiota in RCC progression and treatment response	What is the association between gut microbiota composition and RCC outcomes or therapy efficacy?	To evaluate studies assessing the microbiome’s role in RCC biology and treatment.	Tumor progression markers, treatment response, and immune modulation
Testicular cancer	Psychosocial and mental health outcomes in testicular cancer survivors	What are the long-term psychosocial effects in testicular cancer survivors?	To synthesize evidence on depression, anxiety, and social adjustment post-treatment.	Depression scores, anxiety prevalence, and quality of life

BCG: Bacillus Calmette-Guérin; CSS: cancer-specific survival; OS: overall survival; PFS: progression-free survival; QoL: quality of life; RCC: renal cell carcinoma.

## DISCUSSION

The aim of this research was to validate the ability of ChatGPT to create novel SR questions in urooncology. As AI technology rapidly develops in medicine, the potential of natural LLMs like ChatGPT to assist in scientific research has also become an area of growing interest^17^. SRs are especially important in a wide and ever-changing field such as urooncology, providing valuable advice to researchers and clinicians alike. Consistent with earlier work by Choueka et al.^7^, our research discovered that over half of ChatGPT’s SR recommendations were new.

Methodologically, the model could save time during initial research planning stages through rapid scanning of literature and picking up points with a scarcity of earlier synthesis. Practically, ChatGPT can help identify underrepresented or novel themes—for example, new treatment approaches or understudied patient populations—thus enabling more creative and clinically applicable research questions to be generated^18^. For example, identifying areas where SRs are concentrated in subcategories, such as prostate cancer, kidney tumors, and bladder cancer, can help identify areas where there are still inadequacies. In particular, underrepresented topics like immunotherapy, targeted therapy, and robotic surgery are potentially fertile ground for new questions. Various studies in other fields have used ChatGPT in a similar manner. In their study, Gupta et al.^19^ showed that ChatGPT exhibited an overall accuracy rate of 55% in generating new SR ideas in plastic surgery, in parallel with our study.

The current new idea analysis capability offered by ChatGPT can be useful for urooncology researchers. The model can analyze recent studies and determine which treatment or diagnostic methods are of greater interest. This approach enables the creation of new review topics that require further research beyond the intensively studied areas^20^. For example, underexplored areas of topics such as long-term outcomes of non-surgical treatment approaches in low-risk kidney tumors can be suggested. This ability of ChatGPT offers researchers the chance to generate new ideas by examining the relationships between data, not only by scanning the literature. Similar to the findings of Choueka et al., who identified variability in the accuracy of ChatGPT’s novelty across different subcategories in their urogynecology study, our research also indicated variability among different cancer types, with 70% accuracy for renal tumors and 30% for prostate tumors^7^.

Sensitivity analysis also supported that publication density is significant in the effect it has on noted novelty rates. When novelty was controlled for the saturation of previous SRs, renal cell and testicular cancer had the largest controlled novelty, while prostate and bladder cancers had considerably lower controlled novelty. This analysis shows that ChatGPT’s seeming underperformance in prostate and bladder cancer is to some extent because of publication saturation, rather than a lack of generative ability. Hence, saturation-adjusted novelty better portrays AI performance across heterogeneous subfields. The identical normalization at the GR level also resulted in the same outcomes. RCC and general urooncology exhibited greater adjusted novelty, with prostate, bladder, and testicular cancer exhibiting no or negligible adjusted novelty. It is interesting that with minimal raw novelty, capped off prostate cancer exhibits the capping effect of abundant available literature. On the other hand, the lack of new GR concepts in testicular and bladder cancer reflects possible ChatGPT limitations to hypothesis generation in less served or smaller areas. Collectively, these analyses underscore that novelty aided by AI is field dependent and best utilized in fields with ample but not suffocating saturation of literature.

The main strength of our study is the application of a new, publicly available technology to a new application in urooncology research. To our knowledge, this is one of the first descriptions of the application of ChatGPT in urooncology. We also employed a series of different prompts, general and specific, to test the capacity of ChatGPT to produce new SR concepts. While ChatGPT has shown effectiveness in reviewing literature and pinpointing areas that lack exploration, its ability to generate genuine hypotheses is still constrained^21^. Unlike human researchers, AI systems currently struggle to incorporate subtle clinical reasoning or contextual understanding when developing research queries. Although ChatGPT can propose topics based on trends in existing information, the scientific validity, practicality, and originality of these suggestions still necessitate assessment by experts^14^. Therefore, AI should be regarded not as a substitute for human knowledge but as a complementary tool that can boost efficiency and creativity during the initial stages of research planning. In an effort to address concerns of translational relevance, we piloted a validation trial where five ideas generated by ChatGPT were formulated into structured SR protocols (Table 2). These were found to be sustainable in establishing researchable questions, selecting fit outcomes, and allocating methodological paths. This pilot phase highlights that ChatGPT outputs can transition from abstract concept generation to feasible research proposals under the jurisdiction of expert judgment. Even at this initial stage, this exercise demonstrates the promise of hybrid pipelines whereby generative AI can speed up idea finding and human scientists concentrate on feasibility with clinical relevance and methodological rigor. Subsequent studies will need to be crafted to prospectively evaluate these pilot protocols to assess their effect on research productivity and innovation.

One of the primary shortcomings of the research is the employment of just the PubMed and Scopus databases as a literature source in determining the validity of ChatGPT’s accuracy. We cannot, however, rule out entirely that other studies could have been included had more databases been employed. We conducted this study using ChatGPT 4.0, training it on an October 2023-updated dataset, which presents additional limitations. Relatively few of the studies located through our search of the literature had been published after this date, meaning ChatGPT has limited capability to produce the very latest information. Additionally, we only experimented with a limited set of requests in this study; therefore, the results may not be generalizable to other types of requests, although previous studies have already supported our findings^6,21^.

The second constraint is in the novelty validation process. Sole reliance on bibliographic databases may miss conceptually new research subjects integrated within larger reviews. In addition, our study evaluated the bibliographic novelty of the AI-generated topics based on the absence of prior SRs, but did not explicitly evaluate their scientific quality, feasibility, and clinical priority. Moreover, novelty alone does not imply scientific value, applicability, or clinical significance. Importantly, the aim of the study was not to quantify how many new ideas generated by AI will constitute high-quality or clinically impactful research questions. In our study, expert adjudication was applied to enhance clinical relevance, however, no formal inter-rater reliability measures were calculated; this may limit reproducibility. Notably, this study did not have any formal review of the remaining AI ideas (beyond those five pilot protocols) as to quality and feasibility.

While we hypothesized that ChatGPT’s originality may be inferior to that of human researchers, this was not formally tested. In particular, we lacked a control set of human-generated answers, so direct performance comparison is not possible. We have thus mentioned it as a limitation, and subsequent research should include human benchmarks to better put ChatGPT’s creative capabilities into perspective. That 95%CIs have been supplied helps point to the statistical imprecision in small-sample exploratory research. The broader intervals, as in the general urooncology and renal cell carcinoma, signify variability about observed novelty rates, while the comparatively narrower ones in bladder cancer and prostate cancer signify the few positive findings in these saturated fields. Testicular cancer also had a wide interval, which captures the novel heterogeneity of estimates in underrepresented areas. Reporting CIs thus best conveys the precision of our results, as opposed to point estimates alone.

Highlight the fact that median and IQR analysis were performed only for non-novel concepts, as novel concepts naturally had no pre-existing SRs. This was done to avoid artificially skimming summary statements of the distributions toward zero. Beyond methodological issues, ethical implications of AI-aided research planning must be addressed with specific care. Brought to the forefront in a recent SR, Haltaufderheide and Ranisch demonstrated that the use of ChatGPT and other LLMs in medicine is fraught with transparency, accountability, and bias^10^. Our report on AI use adheres to these guidelines, explicitly detailing the contribution of generative AI in this study. In addition to analyzing novelty rates, we sought to determine if ChatGPT-derived ideas might be formulated into usable research plans. Toward that end, five prototype SR topics, one for each of the urooncologic subspecialties, were molded into formal pilot review protocols (Table 2). Use of pre-established selection criteria guaranteed that only ideas of high clinical utility, conceptual novelty that can be demonstrated, and SR executability were presented to the pilot stage. This systematic approach maximizes the translational value of ChatGPT-generated ideas. The exercise proved that AI-generated ideas can be used practically to formulate clinically relevant research questions. These examples show the capability of generative AI to assist researchers during the initial planning stage and simplify the idea-to-protocol transition. Hybrid workflows of this nature can promote research productivity as well as facilitate research to explore new directions, while expert review remains necessary.

In conclusion, ChatGPT seems like a useful tool to generate novel SR concepts in urooncology. Whereas its identification of literature patterns and proposal of unresearched topics can benefit the pre-exploration research planning phases, its proposals will still require expert validation to remain valid. Far from a replacement for professional insight, ChatGPT might act as a companion that boosts human creativity and effectiveness. As future versions are trained on newer and larger data sets and are incorporated into clinical data systems, these kinds of AI tools might have a greater role in directing high-quality, clinically relevant research.

## Data Availability

The datasets generated and/or analyzed during the current study are available from the corresponding author upon reasonable request.
